# Factors associated with HIV viremia in transgender women and transvestites in five Brazilian capitals, 2019-2021: a multicenter study

**DOI:** 10.1590/S2237-96222024v33e2024412.especial.en

**Published:** 2024-12-06

**Authors:** Claudia Renata dos Santos Barros, Elaine Monteiro Matsuda, Aline Borges Moreira da Rocha, Giselle Ibete Silva López-Lopes, Norberto Camilo Campos, Luís Fernando de Macedo Brígido, Katia Cristina Bassichetto, Maria Amelia de Sousa Mascena Veras

**Affiliations:** 1 Instituto Butantan, Butantã, São Paulo, SP, Brazil; 2 Secretaria Municipal de Saúde de Santo André, Santo André, SP, Brazil; 3 Faculdade de Ciências Médicas da Santa Casa de São Paulo, Departamento de Saúde Coletiva, São Paulo, SP, Brazil; 4 Centro de Virologia, Instituto Adolfo Lutz, São Paulo, SP, Brazil

**Keywords:** Antirretrovirales, Carga Viral, Mujeres Transexuales, Travestis, Estudio Transversal, Antiretroviral therapy, Viral Load, Transsexual Woman, Transvestites, Cross-Sectional Study

## Abstract

**Objective:**

To analyze factors associated with detectable HIV viremia among transgender women/transvestites (TWT) in five Brazilian capitals.

**Methods:**

**:** This was a cross-sectional study using data from a sample of TWT with HIV-positive serology and detectable viral load (VL), between 2019 and 2021. The dependent and independent variables were, respectively: viral load measurement, socioeconomic/demographic characteristics; alcohol/drug use; and self-perceived mental health. Poisson regression with robust variance was used.

**Results:**

**:** A total of 425 TWT tested positive for HIV and underwent VL measurement, 179 (42.0%) presented detectable viremia. Factors positively associated with detectability were: younger age (PR=2.26; 95%CI 1.13;4.51), poorer housing conditions (PR=2.72; 95%CI 1.30;5.68) and poor/very poor mental health (PR=1.70; 95%CI 1.08;2.66). The use of antiretroviral drugs was a protective factor against detectability (PR=0.29; 95%CI 0.30;0.61).

**Conclusion:**

The factors associated with unsuppressed viral load highlight vulnerability related to gender identity that have a negative impact, despite the majority of participants being on antiretroviral therapy (ART).

## Study contributions

**Main results:**

People aged 20–29 who were homeless or unstable and those who reported poor or very poor mental health were more likely to have a detectable viral load. ARV drug use was a protective factor.

**Implications for services:**

The identification of predictors for detectable viral load enables the design of appropriate strategies for monitoring and linking vulnerable people to health services, ensuring effective implementation.

**Perspectives:**

It is necessary to develop educational strategies that take into account important social issues to improve awareness about undetectable viral load in HIV prevention among the transgender population, especially among younger people.

## RESUMEN

**Objetivo:**

Analizar factores asociados a la viremia detectable del VIH entre mujeres transexuales/travestis (MTTr), en cinco capitales brasileñas.

**Métodos:**

Datos referentes al muestreo MTTr con serología reactiva para VIH y carga viral detectable (CV), en estudio transversal entre 2019-2021. Las variables dependientes e independientes fueron, respectivamente: medición de carga viral, socioeconómicas/demográficas; consumo de alcohol/drogas; y autopercepción de salud mental. Se utilizó la regresión de Poisson con varianza robusta.

**Resultados:**

Un total de 425 MTTr dieron positivo para el VIH y se sometieron a pruebas CV, siendo 179 (42,0%) detectables. Los factores asociados positivamente con la detectabilidad fueron: ser más joven (RP=2,26; IC95% 1,13;4,51), peores condiciones de vivienda (RP=2,72; IC95% 1,30;5,68) y salud mental mala/muy mala (RP=1,70; IC95% 1,08;2,66). El uso de medicamentos antirretrovirales fue un factor protector para la detectabilidad (RP=0,29; IC95% 0,30;0,61).

**Conclusión:**

Los factores asociados a la no supresión viral indican vulnerabilidad ligada a identidad de género con efecto negativo, incluso cuando la mayoría utiliza terapia antirretroviral (TARV).

## INTRODUCTION

At the end of 2021, there were approximately 960,000 people living with HIV/AIDS (PLWHA) in Brazil, of whom 852,000 (89.0%) were aware of their HIV infection diagnosis; 800,000 (82%) were linked to a healthcare service; and 730,000 (76.0%) were retained in care, with discontinuation of antiretroviral therapy (ART) or collection of biological samples for monitoring the infection in the last year. In addition, 700,000 (73.0%) were using antiretroviral (ARV) HIV drugs and 627,000 (65.0%) had achieved viral suppression (viral load – VL – less than 50 copies/ mL).^1^ These rates are below the 95-95-95 target set by the Joint United Nations Programme on HIV/AIDS, and were negatively impacted by the COVID -19 pandemic. ^2,3^ It is well acknowledged that most individuals who are diagnosed and properly treated for HIV infection achieve viral suppression and, consequently, elimination of sexual transmission, with the “undetectable ”equals “untransmittable” concept well established, which highlights the importance of treatment as an effective measure in eliminating transmission, serving as a key tool in epidemic control.^4-6^

Studies show that transgender women and transvestite (TWT) represent a group with lower adherence to specialized outpatient care for HIV infection.^5,7,8^ In Brazil, some of the reasons attributed to this population having difficulty accessing and maintaining health care include: (i) being subject to discrimination and prejudice in both public or private healthcare services, making it difficult to retain this population in specialized services for HIV treatment and prevention; (ii) lack of respect for the use of their social names by healthcare professionals in the services they access; (iii) structural violence and, which often results in a higher frequency of alcohol and drug use, higher rates of depression and, consequently, low motivation to adhere to ART, resulting in lower rates of viral suppression; and (iv) poorer social, educational and housing conditions of this population.^9-12^

Given this scenario and the lack of official data through compulsory notification on HIV infection, treatment adherence and viral suppression among TWT, this study estimated the factors associated with detectable HIV viremia among transgender women and transvestites in five Brazilian capitals.

## METHODS

### Study design and location

This was a cross-sectional, multicenter study using data from the TransOdara project, conducted through a mixed-methods approach (quantitative and qualitative), between December 2019 to July 2021, under the coordination of the Faculdade de Ciências Médicas da Santa Casa de São Paulo e do Instituto de Saúde Coletiva da Universidade Federal da Bahia. The project was carried out in five capitals, one in each macro-region of Brazil, namely: São Paulo/São Paulo state (Southeast region), Campo Grande/Mato Grosso do Sul state (Midwest region), Manaus/Amazonas state (North region), Porto Alegre/Rio Grande do Sul state (South region) and Salvador/Bahia state (Northeast region), and aimed to estimate the prevalence of major sexually transmitted infections (STIs), including HIV, viral hepatitis (A, B and C), syphilis and HPV, in addition to asymptomatic and symptomatic infections caused by *Chlamydia trachomatis* and *Neisseria gonorrhoeae* .

Data collection was carried out in person, at pre-defined locations, in each city, for the study. The research workflow included the following tools: eligibility confirmation, questionnaire, acceptability forms for sample collection and pre-consultation procedures, clinical assessment and follow-up form, acceptability forms for sample collection and post-consultation procedures, and laboratory evaluation form.

### Sample

The sample size was calculated based on the objective of estimating the prevalence of syphilis (results of titers >1:8) among transgender women and transvestites, using a design effect of 2. The minimum sample size calculated was 1,280 TWT. Recruitment was conducted using respondent-driven sampling (RDS), a method that selects participants based on their social networks, with this strategy carried out through invitations. Initially, 7-9 “seeds” were selected, and each received five or six invitations, thus initiating the network formation. This method is currently considered one of the most suitable for recruiting hard-to-reach populations.^13^

This study was conducted with a subsample of participants who tested positive for HIV antibodies and underwent a viral load testing.

### Study variables

The dependent variable was the HIV viral load test result (Abbott M2000 HIV PCR-RT), plasma ribonucleic acid (RNA) was extracted using the automated M2000sp instrument and HIV RNA was quantified using real-time polymerase chain reaction (RT-PCR) (Abbott real time M2000rt), according to the manufacturer’s recommendations, with the result expressed in copies/ mL. The test result was categorized as “undetectable” (less than or equal to 50 copies/ mL ) and “detectable” (above this value).

The independent variables were as follows: age group, in years (<20, 20 to 29, 30 to 49, 50 to 68); gender identity (transgender woman, transvestites and others); religion (no religion or belief, Afro-Brazilian, evangelical/protestant, catholic, spiritualist and others); schooling (complete and incomplete elementary school, complete and incomplete high school, complete and incomplete technical high school, complete and incomplete higher education and complete and incomplete postgraduate degree); housing conditions (own or rented house/own or rented apartment, family/friends’ houses, homeless, shelter/boarding house and others); marital status (single, dating/”hooking up”, married/stable union and separated/widowed); occupation (works on the books/retired/public servant, informally employed/self-employed/unemployed/others and sex worker); monthly income, in BRL (up to BRL600; from BRL601 to BRL1,045; from BRL1,046 to BRL1,779; >BRL1,779); lifetime alcohol or illicit drug use (yes/no); self-perception of mental health (good/very good, regular, poor/very poor); and current use of antiretroviral drugs (yes/no).

### Statistical analysis

All variables were described in absolute and relative frequencies. Hypothesis tests for analyzing the differences between the proportions of the independent variables stratified according to the dependent variable (detectable or undetectable) were the Pearson’s chi -square test or Fisher’s exact test.

The analysis of factors associated with detectable HIV viral load was performed using the Poisson regression model with robust variance. Variables associated with the outcome at a significance level of p <0.20 in the bivariate analysis were included, stepwise, in the multiple regression model. In the final model, the variables with p <0.05 and those adjusting by at least 10% to other variables were maintained. The statistical significance level adopted was 5% and the ROC (receiver operating characteristic) was used to analyze the adjustment of the final model. RDS weights were not used in the analysis, as recent studies have shown that they do not improve model performance and may introduce more uncertainty depending on the underlying network structure. Stata 14.1 software was used for analysis.

### Ethical aspects

The project was approved by the Research Ethics Committee of Santa Casa de Misericórdia de São Paulo (CAAE 05585518.7.0000.5479, No. 3.126.815 – 01/30/2019) and other participating institutions, on January 30, 2019. Prior to the interview and biological sample collections, the Free and Informed Consent Form was provided to all study participants.

## RESULTS

Among the 1,317 participants recruited for the study, 425 (32.3%) tested positive for HIV and underwent viral load testing. Of these, the majority were aged between 30 and 49 years (58.8%), identified as transgender women (61.9%), were single (72.1%), and reported either working informally employed or being unemployed (62.6%) at the time of the interview. Half of the study participants (51.9%) had started or completed high school, and 60.6% lived in their own or rented houses or apartments. Monthly income was distributed approximately one-quarter in each category, with an income of up to BRL600 standing out, representing up to half the minimum wage in 2021, based on the value at the time; 30% reported having no religion or belief, and the same proportion reported being Catholic. The majority (77.6%) reported using alcohol or illicit drugs and more than half (52.9%) reported having very good or good mental health, followed by regular mental health. Finally, it could be seen that the majority of participants (87.6%) reported using antiretroviral (ARV) HIV drugs. (Table 1).

**Table 1:** Absolute and relative frequencies and confidence interval of sociodemographicn variables of transgender women and transvestites from five Brazilian capitals (n=425), 2019-2021

**Variables**	**n**	**%**
**Age group (years)**		
<20	8	1, 9
20 to 29	120	28.2
30 to 49	250	58.8
50 to 68	47	11, 1
**Gender** **identity**		
Transgender woman	263	61, 9
Transvestite	153	36.0
Others	9	2.1
**Religion (n=423)** ^a^		
No religion or belief	126	29, 8
Afro-Brazilian	118	27.9
Evangelical/Protestant	34	8.0
Catholic	127	30.0
Spiritist	16	3, 8
Others	2	0.4
**Schooling (n=418)** ^a^		
Complete and incomplete elementary school	135	32.3
Complete and incomplete high school	217	51.9
Complete and incomplete technical high school	15	3.5
Complete and incomplete higher education	45	10, 8
Complete and incomplete postgraduate degree	6	1.4
**Housing conditions** **(n=424)** ^a^		
Own or rented house/apartment	257	60.6
Living with family/friends	108	25, 8
Homeless	13	3.0
Shelter/boarding house	37	8, 8
Others	9	2.1
**Marital status (n=423)** ^a^		
Single	305	72.1
Dating /“hooking up”	53	12.5
Married/stable union	56	13.2
Separated/widowed	9	2.1
**Occupation (n=422)** ^a^		
Works on the books/retired/public servant	50	11, 9
Informally employed/self-employed/unemployed/others	264	62, 6
Sex worker	108	25, 6
**Monthly income (BRL)** **(n=397)** ^a^		
Up to 600	112	28.2
From 601 to 1,045	97	24.4
From 1,046 to 1,779	90	22, 7
>1,779	98	24, 7
**Use of alcohol or illicit drugs**		
No	95	22.3
Yes	330	77.6
**Mental health (self-reported) (n=419)** ^a^		
Good/very good	222	52.9
Regular	153	36.5
Poor/very poor	44	10.5
**Use of antiretroviral drugs (n=299)**		
No	43	12.7
Yes	277	87.2

Among the 425 transgender women and transvestites (TWT) who tested positive for HIV and underwent viral load tests, 179 (42.0%) had detectable results. When comparing the two groups (detectable and undetectable viral load), a higher proportion of individuals with detectable viral load was observed among younger participants (62.5%; p <0.001), those living in poorer housing conditions (69.2%; p <0.001), those who rated their mental health as poor or very poor (52.3%; p=0.013) and those not on ART (89.5%; p <0.01). The other variables did not show statistically significant differences (Table 2).

**Table 2:** Absolute and relative frequencies of sociodemographic variables, alcohol and other drug use and mental health, according to the viral load results of transgender women and transvestites from five Brazilian capitals, TransOdara project, 2019-2021

Variables	Viral load				
	**Undetectable**		**Detectable**		**p-value**
	**n**	**%**	**n**	**%**	
**Age group (years)**					
<20	3	37.5	5	62.5	<0.001
20 to 29	48	40.0	72	60.0	
30 to 49	155	62.0	95	38.0	
50 to 68	40	85.1	7	14.9	
**Gender** **identity**					
Transgender woman	163	61.9	100	38.1	0.059
Transvestite	77	50.3	76	49.7	
Others	6	66, 7	3	33.3	
**Religion**					
No religion or belief	69	54, 6	57	45.4	0.412
Afro-Brazilian	70	59.3	48	40.7	
Evangelical/Protestant	22	64.7	12	35.3	
Catholic	70	55.1	57	44.9	
Spiritist	12	75.0	4	25.0	
Others	2	100	0	0.0	
**Schooling**					
Complete and incomplete elementary school	76	56.3	59	43.7	0.192
Complete and incomplete high school	119	54.4	98	45.6	
Complete and incomplete technical high school	10	66.7	5	33.3	
Complete and incomplete higher education	32	71.1	13	28.9	
Complete and incomplete postgraduate degree	5	83.3	1	16.7	
**Housing conditions**					
Own or rented house/apartment	169	65.8	88	34.2	<0.001
Living with family/friends	52	48.2	56	51.8	
Homeless	4	30.8	9	69.2	
Shelter/boarding house	14	37.8	23	62.2	
Others	6	66.7	3	33.3	
**Marital status**					
Single	172	56.4	133	43.6	0.769
Dating/ “hooking up”	34	64.1	19	35.9	
Married/stable union	33	58.9	23	41.1	
Separated/widowed	5	55.6	4	44.4	
**Occupation**					
Work on the books/retired/public servant	33	66.0	17	34.0	0.346
Informally employed/self-employed/unemployed/others	153	57.9	111	42.1	
Sex worker	58	53.7	50	46.3	
**Monthly income (BRL)**					
up to 600	56	50.0	56	50.0	0.064
601 to 1,045	57	58.8	40	41.2	
1,046 to 1,779	57	63.3	33	36.7	
>1,779	66	67.3	32	32.7	
**Alcohol or illicit drug use**					
No	59	62.1	36	37.9	0.344
Yes	187	56.7	143	43.3	
**Mental health (self-reported)**					
Good/very good	143	64.4	79	35.6	0.013
Regular	78	50.9	75	49.1	
Poor/very poor	21	47.7	23	52.3	
**Use of antiretroviral drugs**					
No	4	10.5	34	89.5	<0.01
Yes	208	79.7	53	20.3	

In the bivariate analysis, it could be seen that being aged between 50 and 68 years old, having a higher monthly income and using ARV drugs were negatively associated with detectable viral load. On the other hand, self-identifying as a transvestite, living with family/friends or being homeless and reporting a perception of mental health as regular, poor or very poor were risk factors for detectable viral load. After adjusting for variables in the multiple analysis, the following remained positively associated with the outcome: being aged between 20 and 29 years (PR=2.26; 95%CI 1.13;4.51), being homeless (PR=2.72; 95%CI 1.30;5.68) or living in a shelter/boarding house (PR=2.15; 95%CI 1.47;3.13) and reporting mental health as poor and very poor (PR=1.70; 95%CI 1.80;2.66). The use of ARV drugs remained a protective factor (PR=0.29; 95%CI 0.13;0.61) (Table 3). The final model showed a good fit, with an ROC explanatory factor of 0.83 (95%CI 0.78;0.89).

**Table 3:** Crude and adjusted prevalence ratio (PR) and confidence interval (95%CI) for sociodemographic variables, alcohol/other drug use and mental health with the detectable viral load results of transgender women and transvestites from five Brazilian capitals, TransOdara project , 2019-2021

	**Bivariate**			**Multiple**		
	**RP**	**95%CI**		**RP**		**95%CI**
**Age group (years)**						
<20	1				1	
20 to 29	0.96	0.55;1.67			2.26	1.13;4.51
30 to 49	0.61	0.35;1.06			1.77	0.88;3.51
50 to 68	0.24	0.19;0.56			0.71	0.24;2.11
**Gender** **identity**						
Transgender woman	1				us	
Transvestite	1.31	1.04;1.63			us	
Others	0.88	0.34;2.23			us	
**Housing conditions**						
Own or rented house/apartment	1				1	
Living with family/friends	1.60	1.21;2.13			1.05	0.72;1.55
Homeless	2.16	1.31;2.58			2.72	1.30;5.68
Shelter/boarding house	1.87	1.31;2.66			2.15	1.47;3.13
Others	1.22	0.48;3.04			1.17	0.28;4.81
**Monthly income (BR** **L)**						
up to 600	1				us	
601 to 1,045	0.82	0.61;1.11			us	
1,046 to 1,779	0.73	0.53;1.02			us	
>1,779	0.65	0.46;0.92			us	
**Occupation**						
Works on the books/retired/public servant	1				us	
Informally employed/self-employed/unemployed/others	1.24	0.82;1.87			us	
Sex worker	1.36	0.88;2.11			us	
**Schooling**						
Complete and incomplete elementary school	1				us	
Complete and incomplete high school	1.03	0.81;1.31			us	
Complete and incomplete technical high school	0.76	0.36;1.6			us	
Complete and incomplete higher education	0.66	0.40;1.08			us	
Complete and incomplete postgraduate degree	0.38	0.63;2.31			us	
**Mental health (self-reported)**						
Good/very good	1				1	
Regular	1.37	1.08;1.75			1.23	0.86;1.74
Poor/very poor	1.47	1.05;2.05			1.70	1.08;2.66
**Use of antiretroviral drugs**						
No	1				1	
Yes	0.23	0.17;0.29			0.29	0.13;0.61

Note: ns : not significant, i.e. p >0.05.

## DISCUSSION

In this study, it could be seen that younger age, unstable housing conditions (boarding house or being homeless) or living with family and friends, and poor or very poor self-perceived mental health were factors associated with detectable levels of viremia. On the other hand, being on antiretroviral therapy, as expected, protected users from having a detectable viral load.

Similar to a study conducted in South Carolina, the United States,^16^ adherence to antiretroviral therapy (ART) was one of the key predictors of achieving and maintaining undetectable viral load, as observed in our findings, through the association between being on ART and lower detectability. Regular use of medication and, consequently, viral suppression are dynamic and multifactorial processes, influenced by individual, social and programmatic characteristics, with emphasis on access to healthcare services.^17,18^ Housing conditions have been found to be a fundamental factor related to the continuity of care for people living with HIV.^12^ Social instability reflected in the lack of stable housing and financial insecurity can hinder the organization required for regular visits to HIV healthcare services and/or dispensing of antiretroviral therapy (ART).

Family and friend support is another important aspect of treatment adherence.^18^ This study did not investigate the relationship between family and friend support and its impact on antiretroviral therapy adherence. However, poorer viremia outcomes were observed among people living with HIV/AIDS (PLWHA) who were homeless or living in temporary housing. Thus, in addition to the social issues involved in these housing conditions, it can be hypothesized that isolation or the lack of close support may play a role. Living with other people can positively impact the health of transgender women when there is support regarding treatment and diagnosis. On the other hand, the negative impact related to stigma on gender identity can be observed, in addition to HIV infection, which is more pronounced among marginalized individuals and in vulnerable situations, resulting in lower adherence and negatively impacting viremia.^19^

The association between older individuals and viral undetectability can be explained by better adherence among individuals with this characteristic, the relationship between time since diagnosis and duration of treatment, acceptance of the diagnosis and understanding of the importance of adherence to treatment for better prognosis and quality of life. Recent diagnosis results in a period of adaptation to treatment and acceptance of one’s condition, which can lead to challenges in medication adherence, negatively impacting viral suppression.^16^

This finding is consistent with other studies that also found a relationship between older age and greater adherence to ART, including among transgender women in São Paulo.^20,21^ However, contrasting with our results, other studies that did not consider gender identity showed poorer adherence among older people, due to higher risks of medication side effects and neurocognitive impairment , which is more prevalent among PLWHA over 50 years of age.^22,23^ A systematic review including 20 studies reported age as a determinant of adherence, with better outcomes observed among older PLWHA (>35 years) compared to younger individuals.^8^ However, this association was not observed in a systematic review and meta-analysis focusing on Africa.^24^ These differences between the present study and the literature highlight the importance of considering both gender and generational issues in analyses of the use of ARV drugs and, consequently, viral load among PLWHA.

Regarding the association between self-assessment of mental health and detectable viremia, our study corroborates research ^25^ that found an association between the presence of common mental disorder and higher viral load in adults living with HIV. Furthermore, mental health is also a predictor of poorer medication adherence, as it may have a direct association with viral load, as well as act as a mediator between adherence to treatment and viremia.^26^ The mechanism linking mental health and viral suppression is bidirectional. Preexisting mental health problem in PLWHA may result in poorer prognosis and treatment adherence. Conversely, the need to cope with comorbidities, or the social impact of HIV diagnosis and treatment, may have negative consequences for mental health.^27^

Mental health issues are further exacerbated among transgender people and transvestites. This population already faces stigma and prejudice related to gender, in addition to poorer socioeconomic conditions.^27^

This study has limitations, such as its cross-sectional design aimed at investigating the prevalence of eight STIs, and it was not specifically designed to assess some issues related to HIV viral suppression. Moreover, the fact that the study was conducted during the COVID-19 pandemic may have influenced the profile of the participating population, which could explain the disproportionately impacted access to care for transgender women and younger participants, as observed in Rio de Janeiro.^3^ However, indicators of care for PLWHA , such as the use of ART and viral suppression rates, were comparable, or even better, among the volunteers in this study, when compared to those of the general population: 77.0% versus 73.0% and 71.6% versus 65.0%, respectively.^1^ We may have accessed a sample with closer ties to healthcare services, higher education levels and older age, which resulted in higher use of ART and viral suppression rates,^2^ than that observed in other studies with similar populations. These studies have shown poorer viral suppression rates and higher loss to follow-up in the transgender population,^3,7^ with better ART adherence results among older transgender women,^20,21^ as younger transgender women may not have been accessed for inclusion in these studies.

Nevertheless, the study included transgender women and transvestites (TWT) from five capital cities – representing a diverse sample of Brazilian macro-regions – and enabled the evaluation of various factors regarding this hard-to-reach population. These findings provide information about a population segment considered vulnerable and neglected, reflecting one of the many expressions of inequality in our society. The leadership of the political and social movement that operates under the acronym LGBTQIAPN+ has contributed to the improvement of public policies, including the expansion of the care networks for people living with HIV/AIDS (PLWHA) in the country.^28^

However, due to prejudice regarding their gender identity, there may have been an increase in the marginalization of this population, as they already face limited access to education, the job market and citizenship. This situation has worsened significantly in recent years, with government actions explicitly targeting sexual and gender minorities were recorded, such as the prohibition of gender-related discussions in schools. These actions have resulted in poorer social indicators, with high rates of external violence, such as homicides and assaults, in addition to psychological suffering, leading to high suicide rates.^29,30^

We concluded that the proportion of PLWHA with detectable viremia was associated with factors related to greater social vulnerability and younger age.

In a country with continental dimensions like Brazil, the findings described here may be even more pronounced in other states and cities not included in this study, which have worse social indicators and, consequently, greater challenges in accessing services and maintaining treatment. The demand for the inclusion of the variable “gender identity” in information systems that monitor the HIV epidemic has not been implemented yet, which has made it difficult to better understand the experiences of the TWT population. We recommend the inclusion of this variable in the information systems of the Brazilian National Health System (Sistema Único de Saúde), accompanied by extensive training of health professionals in this area, so that can inform the development of effective public policies to address inequities observed in this population group.

## References

[1] Brasil (2022). Boletim Epidemiológico.

[2] UNAIDS AE (2023). Relatório Global do UNAIDS.

[3] Bocage AE, Coelho LE, Lake JE, Clark JL, Torres TS, Jalil EM (2023). : Disparities by Age and Gender. AIDS Behav.

[4] Cohen MS, Chen YQ, McCauley M, Gamble T, Hosseinipour MC, Kumarasamy N (2016). Antiretroviral Therapy for the Prevention of HIV-1 Transmission. N Engl J Med.

[5] Rodger AJ, Cambiano V, Bruun T, Vernazza P, Collins S, Degen O (2019). Risk of HIV transmission through condomless sex in serodifferent gay couples with the HIV‐positive partner taking suppressive antiretroviral therapy (PARTNER): final results of a multicenter, prospective, observational study. Lancet.

[6] UNAIDS (2023). Joint United Nations Programme on HIV/AIDS. Understanding Fast-Track: accelerating action to end the AIDS epidemic by 2030.

[7] Heestermans T, Browne JL, Aitken SC, Vervoort SC, Klipstein-Grobusch K (2016). Determinants of adherence to antiretroviral therapy among HIV-positive adults in sub-Saharan Africa: a systematic review. BMJ Glob Health.

[8] Matsuda EM, Oliveira IP, Silva VO, Ahagon CM, Campos IB, Colpas DR (2021). Same week: feasibility of rapid antiretroviral initiation in Brazil. Re:GEN Open.

[9] Lima RRT, Flor TBM, Noro LRA (2023). Systematic review on health care for transvestites and transsexuals in Brazil. Rev Saúde Pública.

[10] Silva JF, Costa GMC (2020). Health care of sexual and gender minorities: an integrative literature review. Rev Bras Enferm.

[11] Radusky PD, Aristegui I, Mandell LN, Dell’Isola E, Zalazar V, Cardozo N (2022). Examining Factors Associated with Gender Identity Among Individuals Disengaged from HIV Care in Argentina. Int J Behav Med.

[12] Pascom ARP, Meireles MV, Benzaken AS (2018). Sociodemographic determinants of attrition in the HIV continuum of care in Brazil, in 2016. Medicine.

[13] Heckathorn DD (1997). Respondent-Driven Sampling: A New Approach to the Study of Hidden Populations. Soc Probl.

[14] Avery L, Rotondi N, McKnight C, Firestone M, Smylie J, Rotondi M (2019). Unweighted regression models perform better than weighted regression techniques for respondent-driven sampling data: results from a simulation study. BMC Med Res Methodol.

[15] Sperandei S, Bastos LS, Ribeiro-Alves M, Reis A, Bastos FI (2023). Assessing logistic regression applied to respondent-driven sampling studies: a simulation study with an application to empirical data. International Journal of Social Research Methodology.

[16] Haider MR, Brown MJ, Harrison S, Yang X, Ingram L, Bhochhibhoya A (2021). Sociodemographic factors affecting viral load suppression among people living with HIV in South Carolina. AIDS Care.

[17] Chiu I, Leathers M, Cano D, Turner CM, Trujillo D, Sicro S (2022). HIV prevalence, engagement in care, and risk behavior among trans women, San Francisco: Evidence of recent successes and remaining challenges. Int J STD AIDS.

[18] Carvalho PP, Barroso SM, Coelho HC, Penaforte FRO (2019). Fatores associados à adesão à terapia antirretroviral em adultos: revisão integrativa de literatura. Cien Saúde Colet.

[19] Techi LC, Cavalcante IS, Lima DA, Oliveira JEM, Lopes SDS, Mendes JPS (2023). Adesão à terapia antirretroviral por pacientes com HIV no Brasil e fatores que a prejudicam: uma revisão integrativa. Pesquisa, Sociedade e Desenvolvimento.

[20] Sabino TE, Avelino-Silva VI, Cavalcantte C, Goulart SP, Luiz OC, Fonseca LAM (2021). Adherence to antiretroviral treatment and quality of life among transgender women living with HIV/AIDS in São Paulo, Brazil. AIDS Care.

[21] Rocha ABM, Barros C, Generoso IP, Bastos FI, Veras MA (2020). HIV continuum of care among trans women and travestis living in São Paulo, Brazil. Rev Saúde Pública.

[22] Silverberg MJ, Leyden W, Horberg MA, DeLorenze GN, Klein D, Quesenberry Jr (2007). CP. Older age and the response to and tolerability of antiretroviral therapy.

[23] Barclay TR, Hinkin CH, Castellon SA, Mason KI, Reinhard MJ, Marion SD (2007). Age-associated predictors of medication adherence in HIV-positive adults: health beliefs, self-efficacy, and neurocognitive status. Health Psychol.

[24] Soomro N, Fitzgerald G, Seeley J, Schatz E, Nachega JB, Negin J (2019). Comparison of Antiretroviral Therapy Adherence Among HIV-Infected Older Adults with Younger Adults in Africa: Systematic Review and Meta-analysis. AIDS Behav.

[25] Nogueira LFR, Pellegrino P, Duarte AS, Inoue SRV, Marqueze EC (2019). Transtornos Mentais Comuns estão associados a maior carga viral em Pessoas Vivendo com HIV. Saúde Debate.

[26] Hou J, Fu J, Meng S, Jiang T, Guo C, Wu H (2020). Posttraumatic Stress Disorder and Nonadherence to Treatment in People Living With HIV: A Systematic Review and Meta-analysis. Front Psychiatry.

[27] Radusky PD, Zalazar V, Cardozo N, Fabian S, Duarte M, Frola C (2020). Reduction of gender identity stigma and improvements in mental health among transgender women initiating HIV treatment in a trans-sensitive clinic in Argentina. Transgender Health.

[28] Oliveira DC (2022). Representativeness of the LGBTQIA+ population in epidemiological research in the context of the National Policy for Comprehensive Health of Lesbians, Gays, Bisexuals, Transvestites and Transsexuals in Brazil: expanding the production of knowledge within the SUS for social justice. Epidemiol Serv Saude.

[29] Benevides BG, Nogueira SNB (2021). Assassinatos e violência contra travestis e transexuais brasileiras em 2020.

[30] Corrêa FHM, Rodrigues BB, Mendonça JC, Cruz LR (2020). Pensamento suicida entre a população transgênero: um estudo epidemiológico. J. Bras. Psiquiatr. [Internet].

