# Supplementary material for: Factors associated with HIV viremia in transgender women and transvestites in five Brazilian capitals, 2019-2021: a multicenter study
Source: Epidemiol Serv Saude. 2024 Dec 6;33(spe1):e2024412. doi: 10.1590/S2237-96222024v33e2024412.especial.en (PMC11654393; doi:10.1590/S2237-96222024v33e2024412.especial.en)
Supplement: Translation

# Translation: Fatores associados à viremia do HIV entre mulheres trans e travestis em cinco capitais brasileiras, 2019-2021: estudo multicêntrico

**DOI:** 10.1590/S2237-96222024v33e2024412.especial.pt

**Authors:** Claudia Renata dos Santos Barros, Elaine Monteiro Matsuda, Aline Borges Moreira da Rocha, Giselle Ibete Silva López-Lopes, Norberto Camilo Campos, Luís Fernando de Macedo Brígido, Katia Cristina Bassichetto, Maria Amelia de Sousa Mascena Veras

**Affiliations:** 1 Instituto Butantan, Butantã, São Paulo, SP, Brasil; 2 Secretaria Municipal de Saúde de Santo André, Santo André, SP, Brasil; 3 Faculdade de Ciências Médicas da Santa Casa de São Paulo, Departamento de Saúde Coletiva, São Paulo, SP, Brasil; 4 Centro de Virologia, Instituto Adolfo Lutz, São Paulo, SP, Brasil

**Keywords:** Antirretrovirais, Carga Viral, Mulher Transexual, Travestis, Estudo Transversal

## Abstract

**Objetivo:**

Analisar fatores associados à viremia detectável de HIV entre mulheres transexuais/travestis (MTTr), em cinco capitais brasileiras.

**Métodos:**

Dados referentes à amostra de MTTr com sorologia reagente para HIV e carga viral (CV) detectável, em um estudo transversal entre 2019 e 2021. As variáveis dependentes e independentes foram, respectivamente: dosagem da carga viral, características socioeconômicas/demográficas; uso de álcool/drogas; e autopercepção de saúde mental. Foi utilizada a regressão de Poisson com variância robusta.

**Resultados:**

Um total de 425 MTTr tiveram resultado reagente para HIV e realizaram dosagem de CV, com 179 (42,0%) detectáveis. Os fatores associados positivamente à detectabilidade foram: serem mais novas (RP=2,26; IC_95_% 1,13;4,51), piores condições de moradia (RP=2,72; IC_95_% 1,30;5,68) e saúde mental ruim/muito ruim (RP=1,70; IC_95_% 1,08;2,66). O uso de antirretroviral foi fator protetor à detectabilidade (RP=0,29; IC_95_% 0,30;0,61).

**Conclusão:**

Os fatores associados à não supressão viral apontam vulnerabilidade ligada à identidade de gênero com impacto negativo, mesmo com a maioria em uso da terapia antirretroviral (TARV).

## Contribuições do estudo

**Principais resultados:**

Pessoas com idade entre 20 e 29 anos que estavam em situação de rua ou instável e aquelas que relataram saúde mental ruim e muito ruim apresentaram maior probabilidade de terem carga viral detectável. O uso de ARV foi fator protetor.

**Implicações para os serviços:**

A identificação de fatores preditores para a carga viral detectável permite desenhar estratégias adequadas para acompanhamento e vinculação das pessoas em situação de vulnerabilidade nos serviços de saúde, de modo a haver uma implementação efetiva.

**Perspectivas:**

É necessário desenvolver estratégias educativas que considerem questões sociais importantes para melhor conscientização sobre a carga viral indetectável na prevenção do HIV entre a população trans, principalmente entre as pessoas mais jovens.

## INTRODUÇÃO

Ao final do ano de 2021, havia aproximadamente 960 mil pessoas vivendo com HIV/aids (PVHA) no Brasil, das quais 852 mil (89,0%) conheciam seu diagnóstico de infecção pelo HIV; 800 mil (82%) estavam vinculadas a algum serviço de saúde; e 730 mil (76,0%), retidas nos serviços, com retirada da terapia antirretroviral (TARV) ou coleta de materiais biológicos para monitoramento da infecção no último ano. Além disso, 700 mil (73,0%) estavam em uso de antirretroviral (ARV) e 627 mil (65,0%) apresentavam supressão viral (carga viral – CV – inferior a 50 cópias/mL).^1^ Essas taxas são inferiores à meta 95-95-95 do Programa Conjunto das Nações Unidas sobre HIV/Aids, e foram impactadas negativamente pela pandemia de covid-19.^2,3^ Sabe-se que a maioria dos indivíduos diagnosticados e tratados adequadamente para a infecção por HIV alcançam a supressão viral e, consequentemente, a eliminação da transmissão por via sexual, com o conceito de “indetectável” igual a “intransmissível” bem estabelecido, o que reforça a importância do tratamento como medida efetiva na eliminação da transmissão, sendo uma ferramenta importante no controle da epidemia.^4-6^

Estudos demonstram que as mulheres trans e travestis (MTTr) configuram um grupo com menor adesão ao seguimento ambulatorial especializado de infecção por HIV.^5,7,8^ No Brasil, entre os motivos atribuídos para que essa população apresente dificuldade de acesso e manutenção de cuidados à saúde estão: (i) serem vítimas de discriminação e preconceito nos serviços de saúde públicos ou privados, dificultando, inclusive, retenção dessa população em serviços especializados para tratamento e prevenção de HIV; (ii) desrespeito ao uso do nome social por parte de profissionais de saúde dos serviços procurados; (iii) violência estrutural e, muitas vezes, em consequência dela, a maior frequência de uso de álcool e outras drogas, maiores índices de depressão e, consequentemente, baixa motivação para o uso da TARV, que se reflete em menores taxas de supressão viral; e (iv) piores condições sociais, educacionais e de moradia dessa população.^9-12^

Diante do cenário apresentado e da escassez de dados oficiais por meio da notificação compulsória sobre infecção pelo HIV, da adesão ao tratamento e da supressão viral entre MTTr, este estudo estimou os fatores associados à viremia detectável do vírus HIV, entre mulheres transexuais e travestis, em cinco capitais brasileiras. 

## MÉTODOS

### Desenho e local do estudo

Este estudo, do tipo transversal e multicêntrico, utilizou as informações do projeto TransOdara, conduzido através de uma abordagem mista (quantitativa e qualitativa), realizada no período de dezembro de 2019 a julho de 2021, sob a coordenação da Faculdade de Ciências Médicas da Santa Casa de São Paulo e do Instituto de Saúde Coletiva da Universidade Federal da Bahia. O projeto foi desenvolvido em cinco capitais, uma em cada macrorregião do Brasil, a saber: São Paulo/SP (região Sudeste), Campo Grande/MS (região Centro-Oeste), Manaus/AM (região Norte), Porto Alegre/RS (região Sul) e Salvador/BA (região Nordeste), e objetivou estimar a prevalência das principais infecções sexualmente transmissíveis (ISTs), entre elas HIV, hepatites virais (A, B e C), sífilis e HPV, além de infecções assintomáticas e sintomáticas por *Chlamydia trachomatis* e *Neisseria gonorrhoeae*. 

A coleta de dados foi realizada presencialmente, em cada um dos locais previamente definidos para a realização do estudo, em cada cidade. Acompanhando o fluxo da pesquisa, foram utilizados os seguintes instrumentos: confirmação de elegibilidade, questionário, formulários de aceitabilidade de coleta e de procedimentos pré-consulta, formulário de avaliação clínica e seguimento, formulários de aceitabilidade de coleta e de procedimentos pós-consulta, e formulário de avaliação laboratorial.

### Amostra

O cálculo amostral do estudo foi conduzido considerando-se o objetivo de estimar a prevalência de sífilis (resultados de títulos >1:8) entre mulheres transsexuais e travestis, utilizando-se o efeito de desenho de 2. O tamanho mínimo calculado da amostra foi de 1.280 MTTr. Para o recrutamento, foi utilizado o *respondent-driven sampling* (RDS), método que considera a seleção das participantes baseada em suas redes sociais, com a estratégia executada mediante convites. Inicialmente, foram selecionadas 7-9 “sementes”, e cada uma recebeu cinco ou seis convites, iniciando-se a construção das redes. Esse método configura-se atualmente como um dos mais apropriados para recrutamento de populações de difícil acesso.^13^

O estudo foi realizado com uma subamostra de participantes com resultado de teste sorológico reagente para detecção de anticorpos anti-HIV e que realizaram exame de carga viral.

### Variáveis do estudo

A variável dependente foi o resultado do exame da carga viral de HIV (Abbott M2000 HIV PCR-RT), o ácido ribonucleico (RNA) plasmático foi extraído no dispositivo automatizado M2000sp e o RNA do HIV foi quantificado utilizando-se a reação em cadeia da polimerase em tempo real (RT-PCR) (*Abbott real time* M2000rt), de acordo com as recomendações do fabricante, com resultado expresso em cópias/mL. O resultado do teste foi categorizado em “indetectável” (menor ou igual a 50 cópias/mL) e “detectável” (acima desse valor). 

As variáveis independentes foram as seguintes: faixa etária, em anos (<20, 20 a 29, 30 a 49, 50 a 68); identidade de gênero (mulher trans, travestis e outras); religião (não tem religião ou crença, afro-brasileira, evangélica/protestante, católica, espírita e outras); escolaridade (fundamental completo e incompleto, médio completo e incompleto, técnico completo e incompleto, superior completo e incompleto e pós-graduação completa e incompleta); condição de moradia (casa própria ou alugada/apartamento próprio ou alugado, residências de família/amigos, situação de rua, abrigo/pensão e outras); situação conjugal (solteira, namorando/“ficando”, casada/união estável e separada/viúva); ocupação (carteira assinada/aposentada/servidora pública, sem carteira assinada/autônoma/desemprega/outras e profissional do sexo); renda mensal, em reais (até R$ 600; de R$ 601 a R$ 1.045; de R$ 1.046 a R$ 1.779; >R$ 1.779); uso de álcool ou drogas ilícitas na vida (sim/não); autopercepção sobre a saúde mental (boa/muito boa, regular, ruim/muito ruim); e estar em uso de antirretrovirais (sim/não). 

### Análises estatísticas

Todas as variáveis foram descritas em frequências absolutas e relativas. Os testes de hipótese para análise das diferenças entre as proporções das variáveis independentes estratificadas, segundo a variável dependente (detectável ou indetectável), foram o teste qui-quadrado e de Pearson ou o teste exato de Fisher. 

A análise dos fatores associados com a carga viral detectável de HIV foi realizada por meio do modelo de regressão de Poisson com variância robusta. As variáveis associadas ao desfecho com nível de significância de p <0,20 na análise bivariada foram inseridas, por *stepwise*, no modelo de regressão múltipla. No modelo final, foram mantidas as variáveis que apresentaram p <0,05 e aquelas variáveis que se ajustaram às demais variáveis em pelo menos 10%. O nível de significância estatística adotado foi de 5% e a curva ROC (*receiver operating characteristic*) foi empregada para análise do ajuste do modelo final. Os pesos RDS não foram utilizados na análise, uma vez que estudos recentes demonstraram que não melhoram o desempenho do modelo e podem introduzir mais incerteza, dependendo da estrutura da rede subjacente. O *software* utilizado para análise foi o Stata 14.1.

### Aspectos éticos

O projeto foi aprovado pelo Comitê de Ética em Pesquisa da Santa Casa de Misericórdia de São Paulo (CAAE 05585518.7.0000.5479, n^o^ 3.126.815 – 30/01/2019) e pelas demais instituições participantes, no dia 30 de janeiro de 2019. Previamente à realização da entrevista e coleta dos materiais biológicos, o Termo de Consentimento Livre e Esclarecido foi apresentado a todas as participantes do estudo.

## RESULTADOS

Entre as 1.317 participantes recrutadas para o estudo, 425 (32,3%) tiveram resultado reagente para HIV e realizaram dosagem de carga viral. Dessas, a maioria tinha idade entre 30 e 49 anos (58,8%), se identificava como mulher transexual (61,9%), estava solteira (72,1%) e relatava estar trabalhando sem carteira assinada ou estar desempregada (62,6%) no momento da entrevista. Metade das participantes envolvidas no estudo (51,9%) tinham iniciado ou concluído o ensino médio, 60,6% residiam em casa ou apartamento próprio ou alugado. A renda mensal apresentou uma distribuição de aproximadamente um quarto para cada categoria, com destaque para a renda de até R$ 600, que representava até meio salário mínimo em 2021, segundo o valor da época; 30% relataram não ter religião ou crença, e a mesma proporção referiu ser católica. A maioria (77,6%) relatou uso de álcool ou drogas ilícitas e mais da metade (52,9%) relatou estar com saúde mental muito boa ou boa, seguida de regular. Por fim, observou-se que a maioria (87,6%) estava em uso de antirretrovirais (ARV), conforme relato das entrevistadas (Tabela 1).

**Tabela 1:** Frequências absolutas, relativas e intervalo de confiança das variáveis sociodemográficas das mulheres transexuais e travestis de cinco capitais do Brasil (n=425), 2019-2021

**Variáveis**	**n**	**%**
**Faixa etária (anos)**		
<20	8	1,9
20 a 29	120	28,2
30 a 49	250	58,8
50 a 68	47	11,1
**Identidade de gênero**		
Mulher trans	263	61,9
Travesti	153	36,0
Outras	9	2,1
**Religião (n=423)** ^a^		
Não tem religião ou crença	126	29,8
Afro-brasileira	118	27,9
Evangélica/protestante	34	8,0
Católica	127	30,0
Espírita	16	3,8
Outras	2	0,4
**Escolaridade (n=418)** ^a^		
Fundamental completo e incompleto	135	32,3
Médio completo e incompleto	217	51,9
Técnico completo e incompleto	15	3,5
Superior completo e incompleto	45	10,8
Pós-graduação completa e incompleta	6	1,4
**Condição de moradia (n=424)** ^a^		
Casa/apartamento próprio ou alugado	257	60,6
Família/amigos	108	25,8
Situação de rua	13	3,0
Abrigo/pensão	37	8,8
Outras	9	2,1
**Situação conjugal (n=423)** ^a^		
Solteira	305	72,1
Namorando/“ficando”	53	12,5
Casada/união estável	56	13,2
Separada/viúva	9	2,1
**Ocupação (n=422)** ^a^		
Carteira assinada/aposentada/servidora pública	50	11,9
Sem carteira assinada/autônoma/desempregada/outras	264	62,6
Profissional do sexo	108	25,6
**Renda mensal (R$)(n=397)** ^a^		
Até 600	112	28,2
De 601 a 1.045	97	24,4
De 1.046 a 1.779	90	22,7
>1.779	98	24,7
**Uso de álcool ou drogas ilícitas**		
Não	95	22,3
Sim	330	77,6
**Saúde mental (autorreferida) (n=419)** ^a^		
Boa/muito boa	222	52,9
Regular	153	36,5
Ruim/muito ruim	44	10,5
**Uso de antirretroviral (n=299)** ^a^		
Não	43	12,7
Sim	277	87,2

Entre as 425 mulheres trans e travestis (MTTr) com resultado positivo para HIV e que dosaram carga viral, 179 (42,0%) apresentaram resultados detectáveis. Ao serem comparados os dois grupos (carga viral detectável e não detectável), observou-se maior proporção de pessoas com carga viral detectável entre as mais jovens (62,5%; p <0,001), que residiam em piores condições de moradia (69,2%; p <0,001), que consideraram a sua saúde mental como ruim ou muito ruim (52,3%; p=0,013) e que não estavam em uso de TARV (89,5%; p <0,01). As demais variáveis não apresentaram diferenças estatisticamente significativas (Tabela 2). 

**Tabela 2:** Frequências absolutas e relativas das variáveis sociodemográficas, uso de álcool e outras drogas e saúde mental, segundo resultado da carga viral de mulheres transexuais e travestis de cinco capitais do Brasil, projeto TransOdara, 2019-2021

**Variáveis**	**Carga viral**				
	**Indetectável**		**Detectável**		**p-valor**
	**n**	**%**	**n**	**%**	
**Faixa etária (anos)**					
<20	3	37,5	5	62,5	<0,001
20 a 29	48	40,0	72	60,0	
30 a 49	155	62,0	95	38,0	
50 a 68	40	85,1	7	14,9	
**Identidade de gênero**					
Mulher trans	163	61,9	100	38,1	0,059
Travesti	77	50,3	76	49,7	
Outras	6	66,7	3	33,3	
**Religião**					
Não tem religião ou crença	69	54,6	57	45,4	0,412
Afro-brasileira	70	59,3	48	40,7	
Evangélica/protestante	22	64,7	12	35,3	
Católica	70	55,1	57	44,9	
Espírita	12	75,0	4	25,0	
Outras	2	100	0	0,0	
**Escolaridade**					
Fundamental completo e incompleto	76	56,3	59	43,7	0,192
Médio completo e incompleto	119	54,4	98	45,6	
Técnico completo e incompleto	10	66,7	5	33,3	
Superior completo e incompleto	32	71,1	13	28,9	
Pós-graduação completa e incompleta	5	83,3	1	16,7	
**Condição de moradia**					
Casa/apartamento próprio ou alugado	169	65,8	88	34,2	<0,001
Na residência de família/amigos	52	48,2	56	51,8	
Situação de rua	4	30,8	9	69,2	
Abrigo/pensão	14	37,8	23	62,2	
Outras	6	66,7	3	33,3	
**Situação conjugal**					
Solteira	172	56,4	133	43,6	0,769
Namorando/“ficando”	34	64,1	19	35,9	
Casada/união estável	33	58,9	23	41,1	
Separada/viúva	5	55,6	4	44,4	
**Ocupação**					
Carteira assinada/aposentada/servidora pública	33	66,0	17	34,0	0,346
Sem carteira assinada/autônoma/desempregada/outras	153	57,9	111	42,1	
Profissional do sexo	58	53,7	50	46,3	
**Renda mensal (reais)**					
até 600	56	50,0	56	50,0	0,064
601 a 1.045	57	58,8	40	41,2	
1.046 a 1.779	57	63,3	33	36,7	
>1.779	66	67,3	32	32,7	
**Uso de álcool ou drogas ilícitas**					
Não	59	62,1	36	37,9	0,344
Sim	187	56,7	143	43,3	
**Saúde mental (autorreferida)**					
Boa/muito boa	143	64,4	79	35,6	0,013
Regular	78	50,9	75	49,1	
Ruim/muito ruim	21	47,7	23	52,3	
**Uso de antirretroviral**					
Não	4	10,5	34	89,5	<0,01
Sim	208	79,7	53	20,3	

Na análise bivariada, observamos que ter idade entre 50 e 68 anos, maior renda mensal e estar em uso de ARV estiveram negativamente associados à carga viral detectável. Por outro lado, se autodeclarar travesti, residir com familiares/amigos ou estar em situação de rua e relatar percepção da saúde mental como regular, ruim ou muito ruim foram fatores de risco para carga viral detectável. Após o ajuste das variáveis na análise múltipla, mantiveram-se associadas positivamente ao desfecho: ter entre 20 e 29 anos (RP=2,26; IC_95_% 1,13;4,51), estar em situação de rua (RP=2,72; IC_95_% 1,30;5,68) ou residir em abrigo/pensão (RP=2,15; IC_95_% 1,47;3,13) e relatar saúde mental como ruim e muito ruim (RP=1,70; IC_95_% 1,80;2,66). O uso de ARV continuou como fator protetor (RP=0,29; IC_95_% 0,13;0,61) (Tabela 3). O modelo final apresentou bom ajuste, com fator explicativo de ROC 0,83 (IC_95_% 0,78;0,89).

**Tabela 3:** Razão de prevalência (RP) bruta e ajustada e intervalo de confiança (IC95%) das variáveis sociodemográficas, uso de álcool/outras drogas e saúde mental com o resultado detectável da carga viral de mulheres transexuais e travestis de cinco capitais do Brasil, projeto TransOdara, 2019-2021

	**Bivariada**			**Múltiplo**		
	**RP**	**IC**_95_ **%**		**RP**		**IC**_95_ **%**
**Faixa etária (anos)**						
<20	1				1	
20 a 29	0,96	0,55;1,67			2,26	1,13;4,51
30 a 49	0,61	0,35;1,06			1,77	0,88;3,51
50 a 68	0,24	0,19;0,56			0,71	0,24;2,11
**Identidade de gênero**						
Mulher trans	1				ns	
Travesti	1,31	1,04;1,63			ns	
Outras	0,88	0,34;2,23			ns	
**Condição de moradia**						
Casa/apartamento próprio ou alugado	1				1	
Família/amigos	1,60	1,21;2,13			1,05	0,72;1,55
Situação de rua	2,16	1,31;2,58			2,72	1,30;5,68
Abrigo/pensão	1,87	1,31;2,66			2,15	1,47;3,13
Outras	1,22	0,48;3,04			1,17	0,28;4,81
**Renda mensal (reais)**						
até 600	1				ns	
601 a 1.045	0,82	0,61;1,11			ns	
1.046 a 1.779	0,73	0,53;1,02			ns	
>1.779	0,65	0,46;0,92			ns	
**Ocupação**						
Carteira assinada/aposentada/servidora pública	1				ns	
Sem carteira assinada/autônoma/desempregada/outras	1,24	0,82;1,87			ns	
Profissional do sexo	1,36	0,88;2,11			ns	
**Escolaridade**						
Fundamental completo e incompleto	1				ns	
Médio completo e incompleto	1,03	0,81;1,31			ns	
Técnico completo e incompleto	0,76	0,36;1,6			ns	
Superior completo e incompleto	0,66	0,40;1,08			ns	
Pós-graduação completa e incompleta	0,38	0,63;2,31			ns	
**Saúde mental (autorreferida)**						
Boa/muito boa	1				1	
Regular	1,37	1,08;1,75			1,23	0,86;1,74
Ruim/muito ruim	1,47	1,05;2,05			1,70	1,08;2,66
**Uso de antirretroviral**						
Não	1				1	
Sim	0,23	0,17;0,29			0,29	0,13;0,61

Nota: ns: não significativo, ou seja, p >0,05.

## DISCUSSÃO

Neste estudo, observou-se que ser jovem, encontrar-se em situação de moradia instável (pensão ou estar em situação de rua) ou na casa de familiares e amigos, e ter percepção de saúde mental ruim ou muito ruim foram fatores associados para viremia em níveis detectáveis. Por outro lado, estar em uso de antirretroviral, como esperado, protegeu as usuárias de apresentarem carga viral detectável. 

Assim como em estudo realizado na Carolina do Sul, Estados Unidos,^16^ a adesão à TARV foi um dos preditores importantes para alcançar e manter a indetectabilidade da carga viral, conforme observado em nossos resultados, por meio da associação entre o uso de TARV e a menor detectabilidade. O uso regular do tratamento medicamentoso e, consequentemente, a supressão viral são processos dinâmicos e multifatoriais, impactados por características individuais, sociais e programáticas, com destaque para o acesso aos serviços de saúde.^17,18^ A condição de moradia tem sido encontrada como um fator fundamental relacionado à continuidade do tratamento de pessoas vivendo com HIV.^12^ A instabilidade social refletida na falta de moradia fixa e a insegurança financeira podem dificultar a organização para visitas regulares a serviços de saúde de HIV e/ou dispensação de ARV. 

O apoio familiar e de amigos é outra questão importante para a adesão ao tratamento.^18^ No estudo, não foi investigada a relação entre apoio familiar e de amigos e seu impacto na adesão ao TARV. No entanto, foram observados piores resultados de viremia entre as pessoas vivendo com HIV/aids (PVHA) que estavam em situação de rua ou residiam em locais temporários. Assim, além de questões sociais envolvidas nessas situações de moradia, podemos hipotetizar sobre o fato do isolamento ou acerca da dificuldade de apoio próximo. A residência com outras pessoas pode impactar positivamente na saúde de mulheres trans, quando há apoio em relação ao tratamento e ao diagnóstico. Por outro lado, pode ser observado o impacto negativo relacionado ao estigma sobre a identidade de gênero, acrescido da infecção pelo HIV, mais evidente entre as pessoas marginalizadas e em situações de maior vulnerabilidade, o que se reflete em menor adesão, com impacto negativo na viremia.^19^

A associação entre as pessoas de maior faixa de idade e indetectabilidade viral pode ser explicada pela melhor adesão entre as pessoas com essa característica, pela relação com o tempo de diagnóstico e o tempo de tratamento, a aceitação do diagnóstico e a compreensão da importância da adesão ao tratamento para melhor prognóstico e qualidade de vida. O diagnóstico recente resulta em período de adaptação para o tratamento e a aceitação da própria condição, fatores que podem resultar em dificuldade à adesão ao tratamento medicamentoso, impactando negativamente na supressão viral.^16^

Esse resultado é concordante com outros estudos que também verificaram essa relação entre maior idade e maior adesão à TARV, inclusive entre mulheres trans de São Paulo.^20,21^ Por outro lado e de forma discordante em relação aos nossos resultados, outros estudos que não consideraram a identidade de gênero apontam a pior adesão entre as pessoas mais velhas, em função do maior risco de efeitos adversos dos medicamentos e pelo comprometimento neurocognitivo, mais prevalente entre PVHA acima dos 50 anos.^22,23^ Em uma revisão sistemática que incluiu 20 estudos, a idade foi relatada como um determinante para a adesão, com melhores desfechos observados entre as PVHAs mais velhas (>35 anos) em comparação às mais jovens.^8^ Tal associação, entretanto, não foi observada em revisão sistemática e metanálise focalizando a África.^24^ Esses contrapontos entre o estudo e a literatura reforçam a importância de se considerarem questões de gênero e geracionais nas análises sobre o uso de ARV e, consequentemente, da carga viral em PVHA.

No que concerne à autoavaliação de saúde mental associada à viremia detectável, nosso estudo corrobora pesquisa^25^ que observou a associação entre a presença de transtorno mental comum e a maior carga viral em adultos vivendo com HIV. Além disso, a saúde mental é também um preditor de pior adesão ao tratamento medicamentoso, uma vez que pode ter uma associação direta com a carga viral, bem como operar como mediação entre a adesão ao tratamento e a viremia.^26^ O mecanismo de associação da saúde mental com a supressão viral é bidirecional. A presença de algum problema relacionado à saúde mental preexistente em PVHA pode resultar em piores prognóstico e adesão ao tratamento. Por outro lado, a necessidade de lidar com as comorbidades, ou lidar socialmente com o diagnóstico e o tratamento para o HIV, pode ter consequências negativas relacionadas à saúde mental.^28^

As questões relativas à saúde mental são ainda mais agravadas entre as pessoas trans e travestis. Essa população já lida com o estigma, com preconceitos por questões de gênero, além de ter piores condições socioeconômicas.^28^

O estudo apresenta limitações, como o desenho transversal voltado à investigação das prevalências de oito ISTs, não tendo sido desenhado especificamente para avaliar algumas questões relativas à supressão viral do HIV. Ademais, o fato de o estudo ter sido desenvolvido durante a pandemia de covid-19 pode ter influenciado no perfil da população participante, o que poderia explicar o acesso aos cuidados ser impactado de forma desproporcional para mulheres trans e participantes mais jovens, como observado no Rio de Janeiro.^3^ Porém, indicadores de assistência às PVHAs, como o uso de TARV e a taxa de supressão viral, foram comparáveis, ou até mesmo melhores, entre as voluntárias deste estudo, quando comparados com os da população geral: 77,0% *versus* 73,0% e 71,6% *versus* 65,0%, respectivamente.^1^ Talvez tenhamos acessado uma amostra que tem mais proximidade com os serviços de saúde, com melhor escolaridade e mais velha, que se refletiu no maior uso de TARV, com maiores taxas de supressão viral^2^ que as observadas em outros estudos com populações semelhantes. Tais pesquisas demonstraram piores taxas de supressão viral e maior perda de seguimento na população trans,^3,7^ com melhores resultados de adesão à ARV entre mulheres trans mais velhas,^20,21^ pois mulheres trans mais jovens podem não ter sido acessadas para inclusão nesses estudos. 

O estudo incluiu mulheres trans e travestis (MTTr) de cinco capitais – uma amostra diversificada das macrorregiões brasileiras –, e possibilitou avaliar diversas informações sobre essa população de difícil acesso. Esses achados fornecem informações sobre um segmento populacional considerado vulnerabilizado e negligenciado, culminando como mais uma das expressões da desigualdade em nossa sociedade. O protagonismo do movimento político e social que opera sob a sigla LGBTQIAPN+ tem contribuído para o aprimoramento de políticas públicas, incluindo-se a ampliação da rede de atenção às pessoas vivendo com HIV/aids (PVHA) no país.^28^

Sobretudo pelo preconceito quanto a sua identidade de gênero, pode ter havido aumento da marginalização dessa população, que já conta com menos acesso à educação, ao mercado de trabalho e à cidadania. Tal cenário se agravou substancialmente nos anos recentes, quando foram registradas ações de governo explicitamente dirigidas contra as minorias sexuais e de gênero, como a proibição de abordar a questão do gênero nas escolas. Tais fatos se refletem em piores indicadores sociais, com altos índices de violência externa, como homicídios e agressões, além de sofrimento psíquico, resultando também em elevada taxa de suicídio.^29,30^

Concluímos que a proporção de PVHA com presença de viremia detectada foi associada a fatores relacionados à maior vulnerabilidade social e menor idade. 

Em um país de dimensões continentais como o Brasil, os achados aqui descritos podem ser ainda mais agravados em outros estados e cidades não incluídas neste estudo, que apresentam piores indicadores sociais, e, consequentemente, podem apresentar mais dificuldades de acesso a serviços e manutenção do tratamento. A reivindicação para a inclusão da variável “identidade de gênero” nos sistemas de informação que monitoram a epidemia de HIV ainda não se viabilizou, o que tem dificultado uma melhor compreensão do que ocorre com a população de MTTr. Recomendamos a inclusão dessa variável nos sistemas de informação do Sistema Único de Saúde, acompanhada de uma ampla capacitação de profissionais de saúde neste entendimento, de modo a termos dados que subsidiem políticas públicas efetivas a serem implementadas para a superação das iniquidades observadas nesse grupo populacional.
